# A Brief History of Cardiac Syndrome X: A Biochemical View

**Published:** 2017-01

**Authors:** Yousef Rasmi, Maryam Majidinia, Fariba Khosravifar, Fatemeh Kheradmand

**Affiliations:** 1 Professor of Clinical Biochemistry, Department of Clinical Biochemistry, Faculty of Medicine, Urmia University of Medical Sciences (UMSU), Urmia, Iran. 5715799313. Tel: +98 44 32770698. Fax: +98 44 33362520. E-mail: rasmiy@umsu.ac.ir.; 2 Student Research Committee, Faculty of Medicine, Tabriz University of Medical Sciences, Tabriz, Iran. 5165665931. Tel: +98 9360488846. Fax: +98 4133364666. E-mail: Majidinia25@gmail.com.; 3 Department of Biology, Tehran Payam-e- Noor University, Tehran, Iran. 19395-4697. Tel: +98 23320000. Fax: +9822441511. E-mail: fkhosravifar1364@gmail.com.; 4 Associated Professor of Clinical Biochemistry, Department of Clinical Biochemistry, Faculty of Medicine, Urmia University of Medical Sciences (UMSU), Urmia, Iran. 5715799313. Tel: +98 44 32770698. Fax: +98 44 33362520. E-mail: fkheradmand@yahoo.com.

When coronary angiography became more widely used in the 1960s, it was soon apparent that not all patients with clinical suspicion of coronary artery diseases had obstruction of the epicardial coronary arteries.^1^ In 1967, Likoff et al.^2^ reported on 15 women ranging in age from 30 to 53 years with chest pain despite normal coronary angiograms, but with electrocardiogram (ECG) abnormalities at rest (ST-segment depression or T-wave inversion) accentuated by exercise. It was the starting point of the story. 

Six years later, in 1973, Kemp et al.^3^ published the 1st definitive study and termed this disease “syndrome X” to denote the uncertainty of the chest pain etiology in these patients. This term was subsequently used by other investigators but often with different criteria as to its definition. 

Cardiac syndrome X (CSX) or microvascular angina is a clinical definition that refers to patients with 3 features: 1) chest pain, 2) positive stress test and ST-segment depression, and 3) normal coronary arteries on angiography.^1^^, ^^4^

The pathological mechanism responsible for CSX is not clearly understood, in spite of extensive studies. Endothelial dysfunction,^5^ myocardial ischemia,^6^ abnormal pain perception, infection,^7^ and estrogen deficiency are among the most commonly suggested pathogenic mechanisms.^8^ Given the high frequency of *Helicobacter pylori* (*H. pylori*) infection in CSX and the probable causative effect of chronic infection in vascular disease, *H. pylori* has a probable role in the pathogenesis of CSX.^9^ In 2009, we hypothesized that *H. pylori *infection could be a trigger for the probable mechanism of endothelial dysfunction via chronic inflammation in the pathogenesis of CSX.^10^

Several published series have reported that some of the patients who undergo coronary angiography for the investigation of chest pain are found to have CSX.

In 1981, Opherk et al.^11^ reported a limited increase in the coronary blood flow (CBF) after dipyridamole infusion in 21 normotensive nondiabetic patients with CSX by comparison to 15 controls with atypical chest pain unresponsive to nitrates and with normal ECG responses to exercise. In addition, in 1983, Cannon et al.^12^ reported limitation in the great cardiac vein flow response to rapid atrial pacing in patients who described their typical chest pain as being provoked by this stress. After 5 years, Cannon and Epstein^13^ proposed the term “microvascular angina” for this patient population for the 1st time in view of what appeared to be heightened sensitivity of the coronary microcirculation to vasoconstrictor stimuli associated with a limited microvascular vasodilator capacity.

The recognition that many patients with chest pain and angiographically normal coronary arteries do not have convincing evidence of CSX has led several groups to consider abnormal pain perception as a fundamental abnormality in this patient population. In 1987, Turiel et al.^14^ reported that patients with typical angina, normal coronary angiograms, and ischemic-appearing ECG responses to exercise had a lower pain threshold and tolerance for forearm ischemia and electrical skin stimulation than patients with coronary artery disease. In 1991, Camici et al.^15^ assessed myocardial lactate production in patients with typical angina pectoris and normal angiograms who had chest pain and ischemic-appearing ECG changes during exercise stress and found no evidence for it. Many investigations were done in the same year. For example, Dean et al.^16^ reported insulin resistance in a small series of patients with chest pain and normal coronary angiograms because of abnormal myocardial handling of glucose and lactate. Additionally, endothelial dysfunction was introduced as a pathological mechanism for CSX and was suggested by Motz et al.,^17^ who assessed the CBF response to intracoronary acetylcholine and to intravenous dipyridamole in such patients. In a subsequent study, Egashira et al.^18^ showed that the injection of increasing doses of acetylcholine into the left coronary artery caused a lower dose-dependent increase in the CBF in patients with CSX. Thereafter in 1995, Kaski et al.^8^ demonstrated increased blood concentrations of endothelin, which showed the presence of abnormal endothelial function in these patients. On the other hand, Rosano et al.^19^ confirmed that estrogen deficiency was associated with CSX and ovarian hormone deficiency might, therefore, play a role in the onset of syndrome X in female patients.

17β‐estradiol attenuates the production of adenosine and modulates its actions.^20^^, ^^21^ The alleviation of chest pain by estrogen, found in some studies, does not necessarily imply an anti‐ischemic action.^21^ Estrogen replacement therapy *per se *rarely results in a meaningful improvement of ischemic ECG changes. Estrogen type, variable combinations of progestogens and estrogen, and timing of administration may be responsible for the lack of more clear-cut results with the use of the stated agents. Different pathogenic mechanisms operating in various patients may also be responsible for the lack of efficacy of estrogen in some trials but not in others. However, estrogen administration often is a useful adjunctive therapy in subgroups of women with CSX, particularly those with angina symptoms associated with variations in hormonal values.^22^

Also, it has been reported that patients with the angiographic exclusion of a coronary artery disease have a significantly increased incidence of mental symptoms compared to the healthy population. Therefore, a psychosomatic diagnostic should be performed early in these patients.  The investigators have recommended the use of 3 standard questionnaires as initial screening tools for mental symptoms. These diagnostic tools may prevent repeated utilization of the healthcare system and this could help to reduce costs for these patients due to the initiation of an early psychosomatic therapy.^23^

Koren et al.^24^ in 1997 stated that microvascular angina was associated with enhanced Na^+^/H^+^ exchange in erythrocytes, probably due to more extensive phosphorylation of the membrane antiporter sites. In the same year, Lanza et al.^6^ indicated an abnormal cardiac adrenergic nerve function, which strongly supported the cardiac origin of the chest pain in syndrome X. Tousoulis et al.^25^ in 2001 showed that intercellular adhesion molecule-1 (ICAM-1) and vascular cell adhesion molecule 1 (VCAM-1) levels were increased in patients with syndrome X. These findings suggest the presence of chronic inflammation with the involvement of the endothelium in these patients. Moreover, Lanza et al.^26^ stated that platelet agreeability at rest was increased in syndrome X and was reduced by exercise. This response was prevented by theophylline, strongly suggesting the involvement of adenosine. In 2002, Altun et al.^27^ declared that in these patients nocturnal melatonin synthesis decreased markedly because of downregulation in beta-adrenergic receptors due to excessive sympathetic activation. On the other hand, Ščudlová et al.^28^ in 2003 concluded that the spastic phenomenon in the coronary artery circulation was not necessarily accompanied by endothelial dysfunction. This supports the hypothesis that the microvascular form of angina is the early stage of coronary artery atherosclerosis. In the same year, Cosin-Sales et al.^29^ reported that C-reactive protein values correlated with symptoms and ECG markers of myocardial ischemia in these patients and, thus, suggested that inflammatory mechanisms might be responsible, at least in part, for endothelial dysfunction and increased disease activity in patients with CSX. Recently, we reported that metoprolol improved endothelial function and suggested that it might be deemed a choice for CSX treatment. It seems that beta-blockers may constitute a choice for the treatment of CSX. Accordingly, it can be concluded that improvement in endothelial function is one of the treatment lines for patients with CSX.^30^

In 2007, a polymorphism case was reported for CSX by Jabrocka-Hybel et al.^31^ Suggesting that the polymorphisms of the angiotensin-converting enzyme gene and endothelial nitric oxide synthase gene was associated with left ventricular hypertrophy, the authors assessed the association between polymorphisms and left ventricular hypertrophy in patients with CSX and concluded that the presence of allele 4 of endothelial nitric oxide synthase (eNOS) gene intron 4 VNTR polymorphism could predispose to cardiac hypertrophy.

In 2013, a large number of papers published their new results on the mechanism of CSX.

These papers can be divided into 2 groups. In the 1st group, some new proteins were assessed in CSX. For example, Celik et al.^32^ found that adropin levels, which affect the regulation of endothelial function, was significantly lower in CSX patients and concluded that adropin was an independent risk factor for CSX. In another study, Buyukkaya et al*.*^33^ introduced a new inflammatory factor, termed “pentraxin-3”, which is an acute phase reactant and resembles C-reactive protein structurally and functionally. The investigators measured its levels in 122 patients and found significant elevations in CSX patients.

In the 2nd group, gene polymorphism in CSX was investigated. For instance, Sinici et al.^34^ investigated intron 4 VNTR polymorphism of the eNOS gene in these patients and concluded that intron 4aa genotype of the eNOS gene was protective for CSX. In another study, Güler et al.^35^ surveyed Factor XIII Val34Leu polymorphism in these patients and found that the frequency of Factor XIII (valine/leucine [Val/Leu] + leucine/leucine [Leu/Leu]) mutation was significantly higher in patients with CSX than in controls. 

What has been delineated thus far, far from being the end of the journey, is merely a faint light on the biochemical pathways of CSX.
